# Supplementary material for: COVID-19 Clusters in Belgian Nursing Homes: Impact of Facility Characteristics and Vaccination on Cluster Occurrence, Duration and Severity
Source: Viruses. 2023 Jan 13;15(1):232. doi: 10.3390/v15010232 (PMC9867491; doi:10.3390/v15010232)
Supplement: Supplementary file 1 [file viruses-15-00232-s001.zip › viruses-2097834-supplementary/Table S1.pdf]

Supplementary Table S1. Variables calculated based on surveillance data for each possible cluster in nursing homes, June 22, 2020 to January 2, 2022, Belgium.

**Variables**	**Description**
Characteristics of the institution	unique identification number, region, number of beds, number of occupied bed (number of beds occupied on that day including beds occupied by hospitalized residents and short-stay residents), number of staff (all personnel working in the facility, including nursing staff, paramedical staff, animation team, staff concerned with cleaning, maintenance or quality control, and NH managers and their administrative staff, but excluding students and volunteers) and type of institution.
Data about the current cluster	start date, end date, number of days the cluster lasted, number of confirmed cases among residents during the cluster (cumulative number of newly reported cases between the start date and the end date), number of confirmed cases among staff during the cluster (cumulative number of newly reported cases between the start date and the end date), COVID-19 wave in which the cluster occurred.
Number of clusters that occurred within the institution within the study period	from 22/06/2020 until 02/01/2022 included.
Data about the vaccination campaign *of the primary course*	date first vaccination dose (in the institution), date second vaccination dose (in the institution), vaccination rate fully vaccinated residents at the end of the vaccination campaign, vaccination rate fully vaccinated staff members at the end of the vaccination campaign.
Previous cluster	to calculate if the NH had a cluster during W1, the prevalence numbers of the surveillance data were used. When the nursing home reported a prevalence of at least two possible or confirmed cases during W1, this was seen as a previous possible cluster. For the other waves, the number of clusters within the nursing home was used to calculate if they had a cluster before.
