# Supplementary material for: COVID-19 Clusters in Belgian Nursing Homes: Impact of Facility Characteristics and Vaccination on Cluster Occurrence, Duration and Severity
Source: Viruses. 2023 Jan 13;15(1):232. doi: 10.3390/v15010232 (PMC9867491; doi:10.3390/v15010232)
Supplement: Supplementary file 1 [file viruses-15-00232-s001.zip › viruses-2097834-supplementary/Table S2.pdf]

Supplementary Table S2. Univariate negative binomial regression of factors influencing the cluster characteristics (size, hospital admissions, deaths, duration) (negative binomial regression) for the period from June 22, 2020 to January 2, 2022, Belgium.

	**Cases**	**Hospital admissions**	**Deaths**	**Duration of clusters (days)**
	Estimate (Wald 95% CI)	Estimate (Wald 95% CI)	Estimate (Wald 95% CI)	Estimate (Wald 95% CI)
Ownership				
Public	Ref.	Ref.	Ref.	Ref.
Private nonprofit	-0.003	-0.15	0.13	**-0.05****
	(-0.11 – 0.11)	(-0.34 – -0.04)	(-0.06 – 0.31)	**(-0.11 – -0.001)**
Private for-profit	-0.10*	-0.13	**-0.39*****	**-0.06 ****
	(-0.22 – 0.02)	(-0.32 – 0.07)	**(-0.58 – -0.19)**	**(-0.11 – -0.004)**
Size				
Small	**-0.15****	-0.05	-0.08	**-0.10*****
	**(-0.24 – -0.06)**	(-0.20 – 0.11)	(-0.24 – 0.07)	**(-0.14 – -0.05)**
Large	Ref.	Ref.	Ref.	Ref.
Region				
Flanders	Ref.	Ref.	Ref.	Ref.
Brussels	**-0.004****	**0.25****	**-0.80*****	-0.04
	**(-0.42 – -0.11)**	**(0.08 – 0.41)**	**(-1.07 – -0.54)**	(-0.11 – 0.04)
Wallonia	-0.26	**-0.34****	**-0.74*****	-0.04
	(-0.42 – -0.11)	**(-0.61 – -0.06)**	**(-0.90 – -0.58)**	(-0.05 – 0.04)
Wave				
Interwave 1	Ref.	Ref.	Ref.	Ref.
2nd wave	**1.37*****	**0.98*****	**1.42*****	**0.45*****
	**(1.12 – 1.62)**	**(0.53 – 1.42)**	**(0.99 – 1.85)**	**(0.33 – 0.58)**
3th wave	0.18	-0.41	-0.48*	**0.20****
	(-0.11 – 0.47)	(-0.93 – 0.12)	(-0.99 – 0.03)	**(0.05 – 0.34)**
Interwave 2	**0.38****	-0.40	**-0.61****	**0.41*****
	**(0.07 – 0.68)**	(-0.95 – 0.16)	**(-1.16 – -0.06)**	**(0.25 – 0.56)**
4th wave	**0.35****	-0.41*	**-0.61****	**0.39*****
	**(0.08 – 0.62)**	(0.88 – 0.07)	**(-1.16 – -0.06)**	**(0.26 – 0.52)**
5th wave	**0.97*****	**-1.32****	**-1.59*****	**0.72*****
	**(0.60 – 1.35)**	**(-2.17 – -0.48)**	**(-2.47 – -0.71)**	**(0.53 – 0.91)**
Previous cluster				
No	Ref.	Ref.	Ref.	Ref.
Yes	**-0.39*****	**-0.54*****	**-0.40*****	-0.06*
	**(0.53 – -0.26)**	**(-0.76 – -0.32)**	**(-0.63 – -0.18)**	(-0.12 – 0.005)
Start vaccination				
Vaccination not started	Ref.	Ref.	Ref.	Ref.
First dose given	**-0.59*****	**-0.87*****	**-0.70*****	**-0.19*****
	**(-0.79 – -0.38)**	**(-1.23 – -0.50)**	**(-1.02 – -0.36)**	**(-0.30 – -0.09)**
Second dose given	**-0.95*****	**-1.37*****	**-1.96*****	-0.06
	**(-1.04 – -0.86)**	**(-1.54 – 1.20)**	**(-2.13 – -1.79)**	(-0.10 – -0.01)

*P<0.1, **P<0.05, ***P<0.001
